# Supplementary figures and images for: A novel broad host range phage phiA85 displays a synergistic effect with antibiotics targeting carbapenem-resistant Klebsiella pneumoniae
Source: Microbiol Spectr. 2025 Aug 15;13(10):e02019-25. doi: 10.1128/spectrum.02019-25 (PMC12502666; doi:10.1128/spectrum.02019-25)

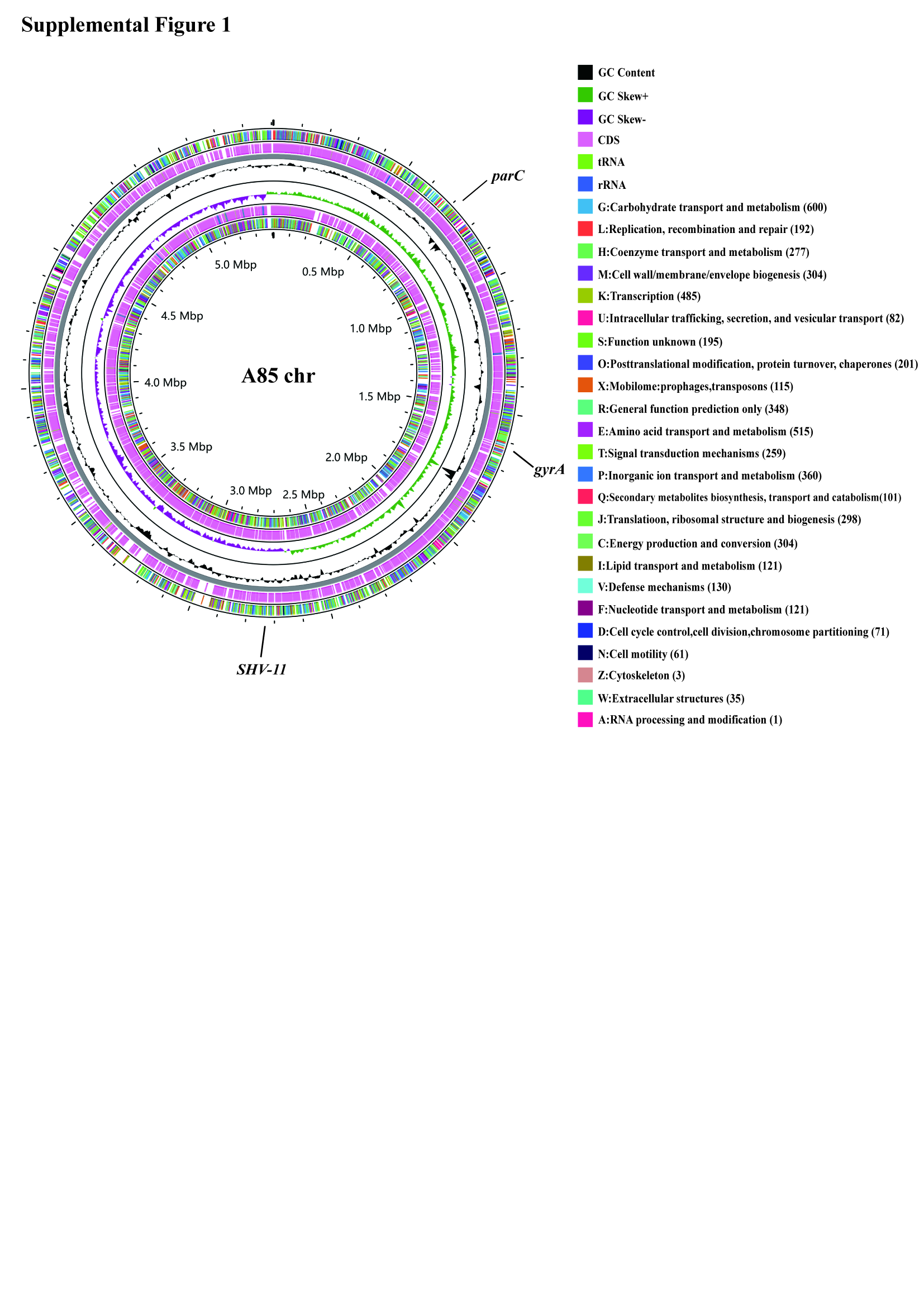

Supplement: Fig. S1 — The genomic map of the chromosome of A85 (A85 chr). [file spectrum.02019-25-s0001.tif]

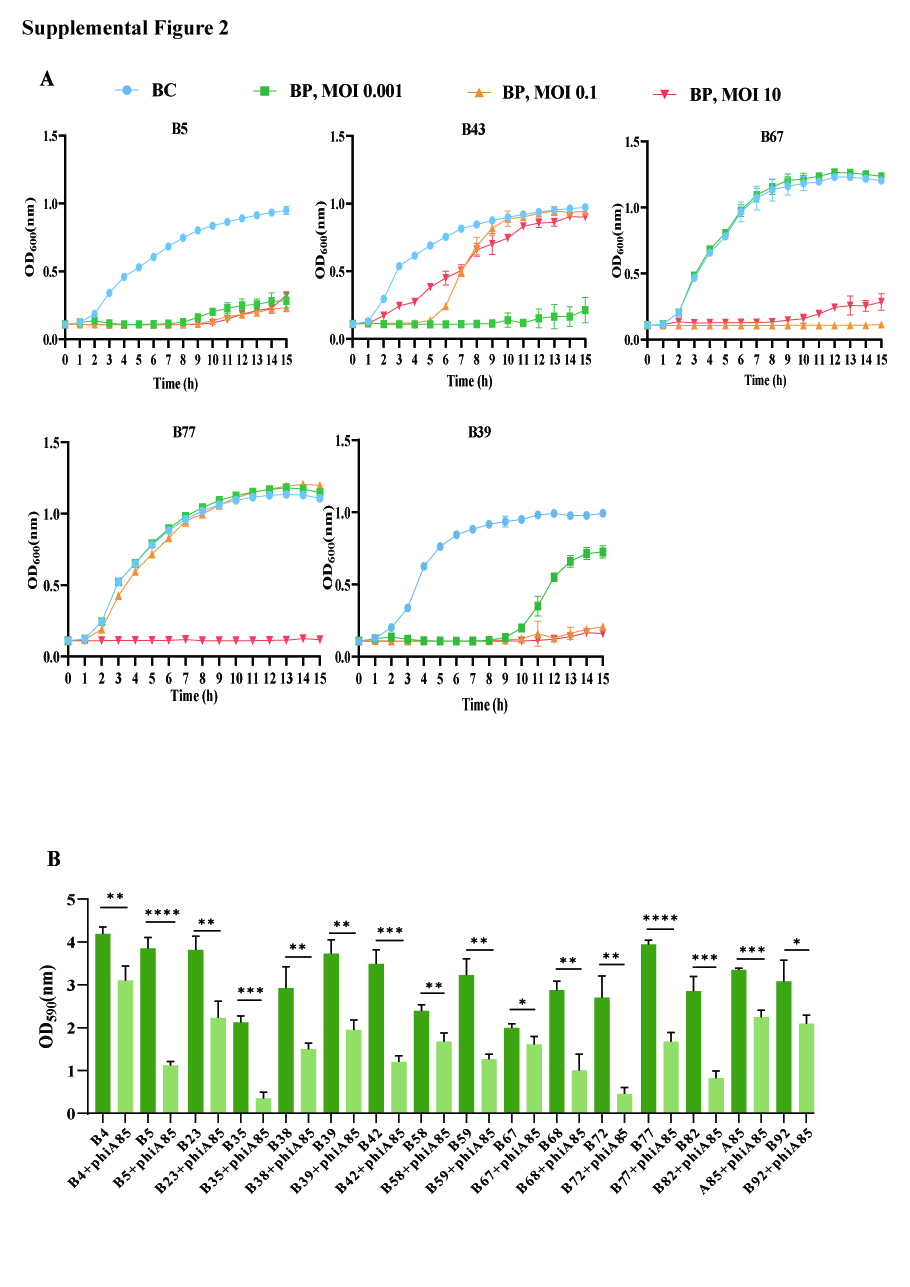

Supplement: Fig. S2 — A. Killing curves of phage phiA85 against host strains. B. The inhibitory effect of the phiA85 on biofilm formation of host strains. [file spectrum.02019-25-s0002.tif]

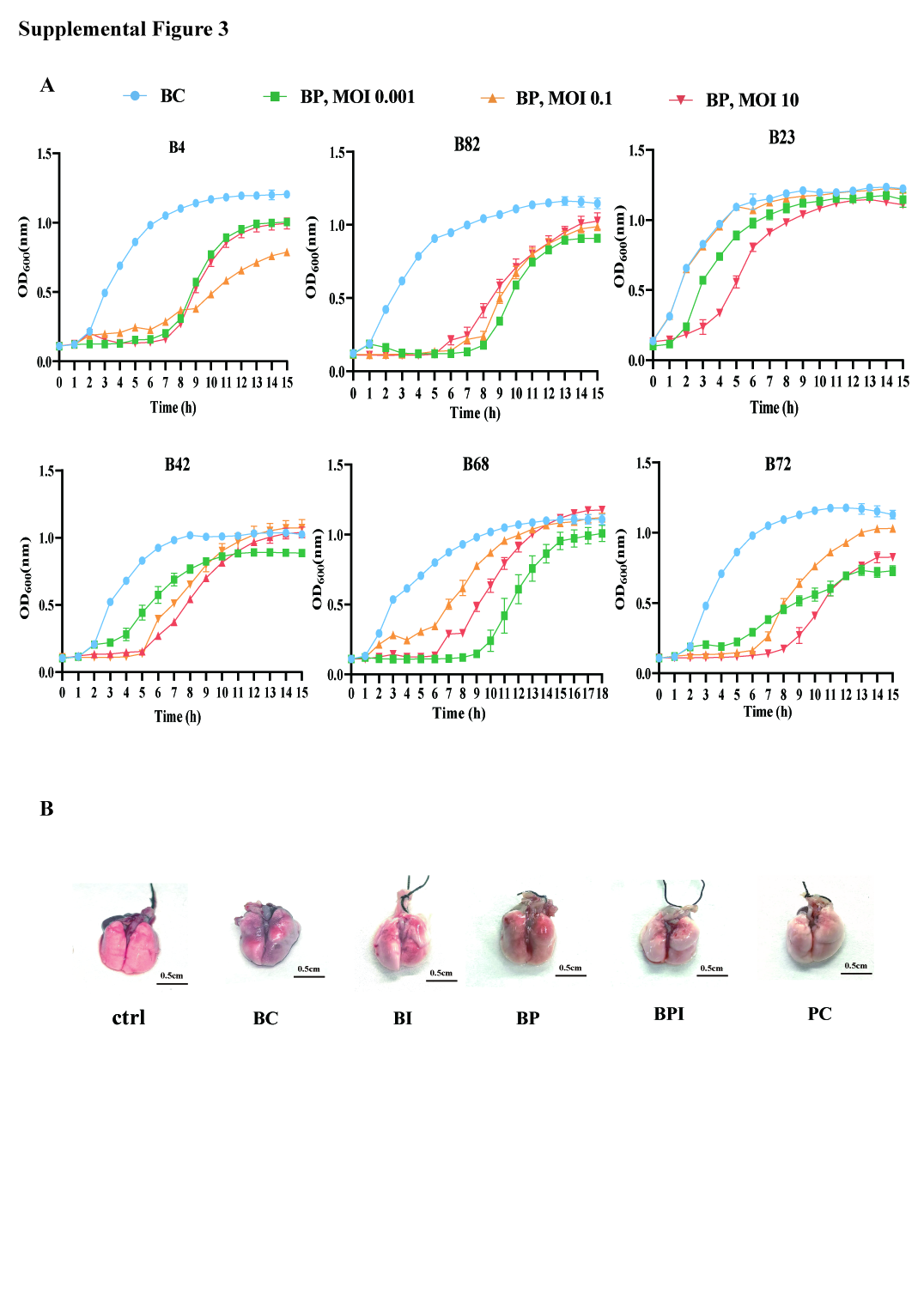

Supplement: Fig. S3 — A. Killing curves of phage phiA85 against host strains. B. Anatomy images of mice lungs in different groups. [file spectrum.02019-25-s0003.tif]

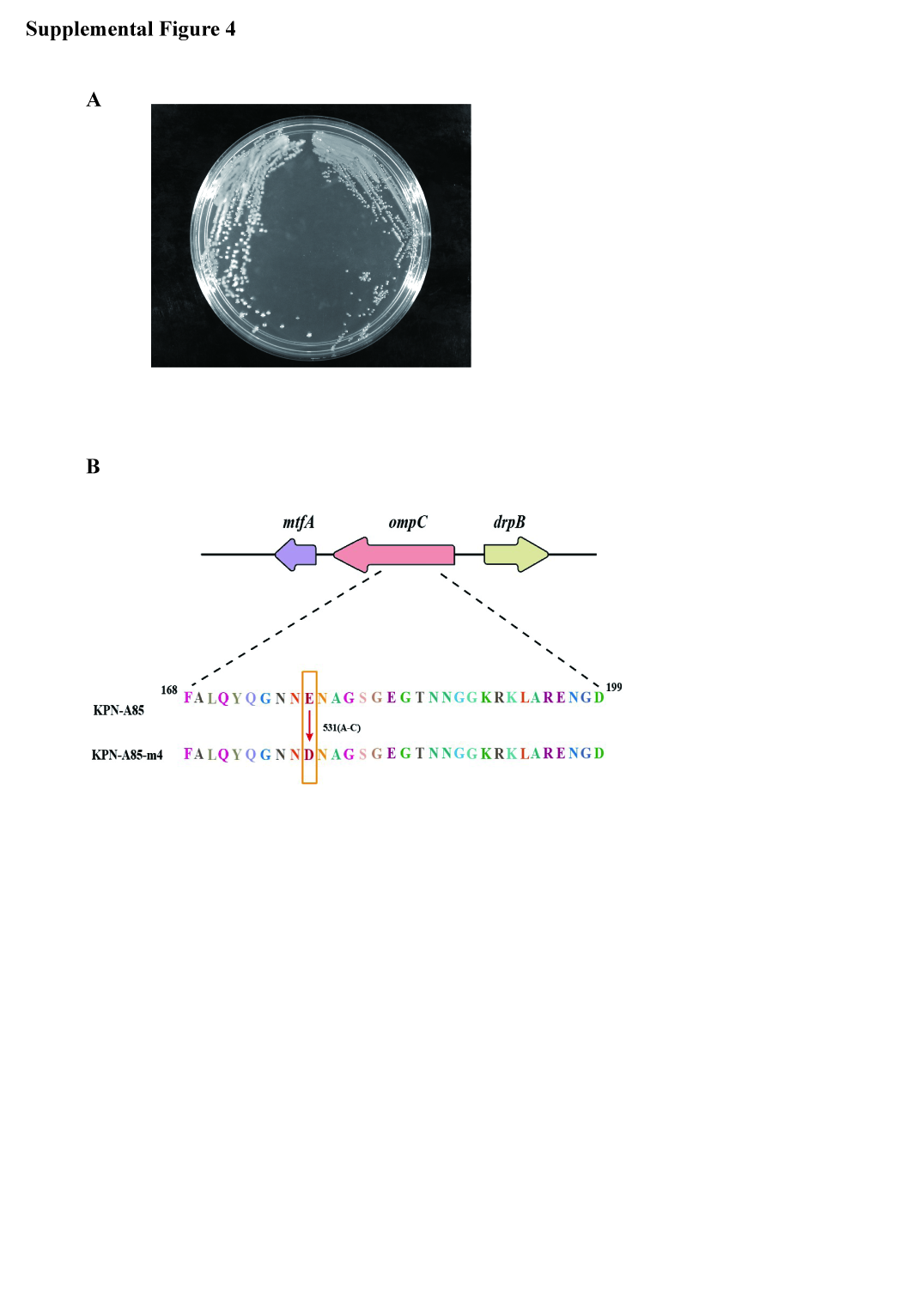

Supplement: Fig. S4 — A. The colony morphology of A85 (left) and its phage mutant strains (right). B. Schematic diagram of phage mutant mutation sites. [file spectrum.02019-25-s0004.tif]
