# Supplementary material for: A novel broad host range phage phiA85 displays a synergistic effect with antibiotics targeting carbapenem-resistant Klebsiella pneumoniae
Source: Microbiol Spectr. 2025 Aug 15;13(10):e02019-25. doi: 10.1128/spectrum.02019-25 (PMC12502666; doi:10.1128/spectrum.02019-25)
Supplement: Supplemental material — Supplemental figure legends. [file spectrum.02019-25-s0005.docx]

**Figure Legends**

**Supplemental Figure 1**

The genomic map of the chromosome of A85 (A85 chr). From the innermost to the outermost, the concentric circles represent: Genome scale (base pair); COG (Clusters of Orthologous Groups) locations on the negative strand; Coding sequences (CDS), tRNA, and rRNA positions on the negative strand; GC Skew; GC Content; CDS, tRNA, and rRNA positions on the positive strand; COG locations on the positive strand.

**Supplemental Figure 2**

**A.** Killing curves of phage phiA85 against host strains. BC represents bacteria control. BP means bacteria and phage mixture in different MOIs. Data represent mean ± s.d. (n=3). **B.** The inhibitory effect of the phiA85 on biofilm formation of host strains. Data represent mean ± SD (n=3). Statistical significance was determined using Student's *t*-test. **P* < 0.05, ***P* < 0.01, ****P* < 0.001, *****P* < 0.0001, ns, not significant.

**Supplemental Figure 3**

**A.** Killing curves of phage phiA85 against host strains. BC represents bacteria control. BP means bacteria and phage mixture in different MOIs. Data represent mean ± SD (n=3). **B.** Anatomy images of mice lungs in different groups.

**Supplemental Figure 4**

**A.** The colony morphology of A85 (left) and its phage mutant strains (right). **B.** Schematic diagram of phage mutant mutation sites.
